# Supplementary material for: β-amyloid and tau drive early Alzheimer’s disease decline while glucose hypometabolism drives late decline
Source: Commun Biol. 2020 Jul 6;3:352. doi: 10.1038/s42003-020-1079-x (PMC7338410; doi:10.1038/s42003-020-1079-x)
Supplement: Supplementary file 1 — Supplementary Information [file 42003_2020_1079_MOESM1_ESM.pdf]

Supplementary Information

# β-Amyloid and Tau Drive Early Alzheimer's Disease Decline While Glucose Hypometabolism Drives Late Decline

Data used in preparation of this article were obtained from the Alzheimer's Disease Neuroimaging Initiative (ADNI) database (adni.loni.usc.edu). As such, the investigators within the ADNI contributed to the design and implementation of ADNI and/or provided data but did not participate in analysis or writing of this report. A complete listing of ADNI investigators can be found at the end of the supplementary information.

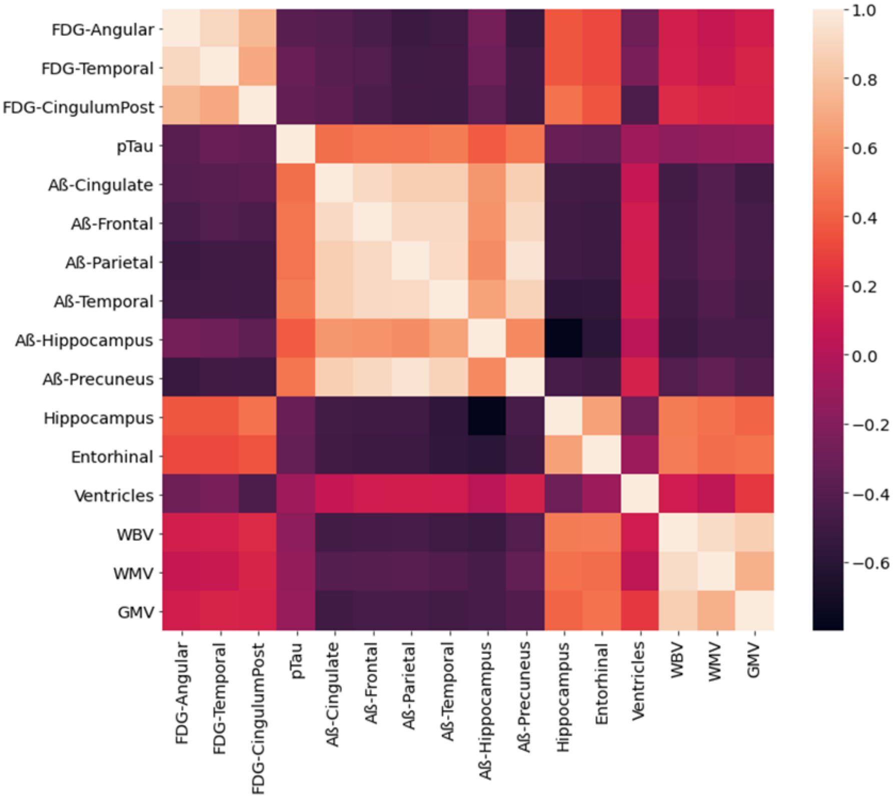


**Supplementary Figure 1. Correlation Heatmap of biomarker features.** Heatmap depicting how the biomarker features were correlated one with another. The color legend is representative of pearson correlation r values.

**CU vs. LMCI:**

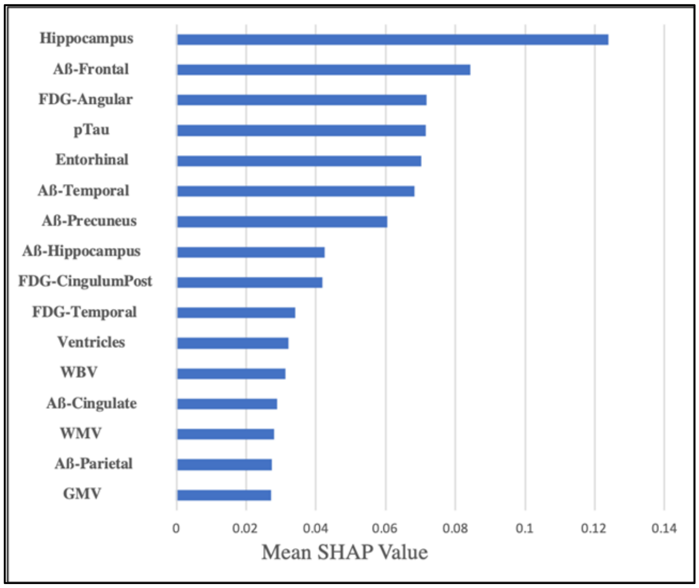


**LMCI vs. AD:**

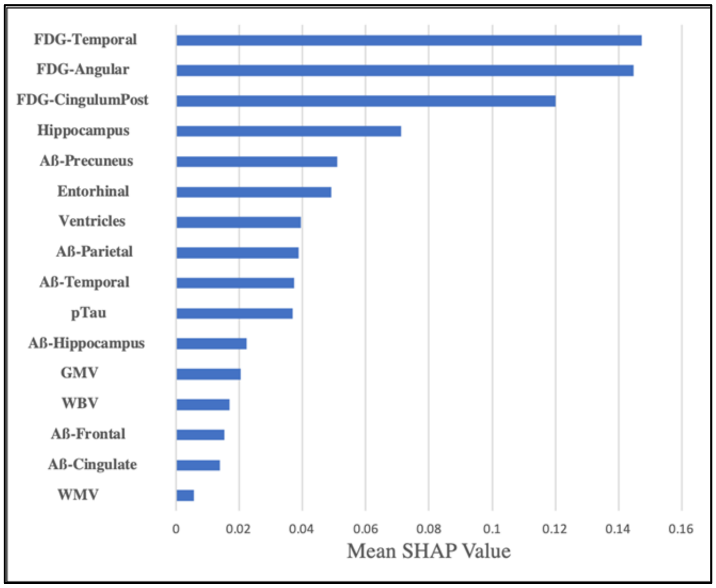

**CU vs. AD:**

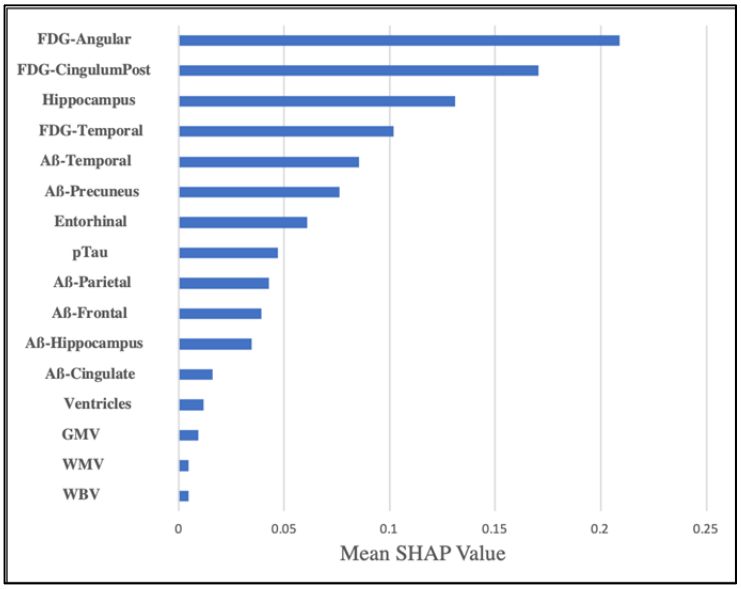


**Supplementary Figure 2. SHAP Analysis depicting biomarker feature ranking importance.** Comparison of feature ranking analysis from implementation of the SHapley Additive exPlanations (SHAP) technique. The bar plots shows feature impacts on the cognitively unimpaired (CU) vs late mild cognitive impairment (LMCI) analysis, the LMCI vs Alzheimer’s disease (AD) analysis, and the CU vs AD analysis.

**Supplementary Table 1. Accuracy results of all features and Top 8 features in Random Forest model from A) three- and B) ten-fold cross validation.**

A) K-fold=3

| All Features | | | |
|---|---|---|---|
| | CU vs. LMCI | LMCI vs. AD | CU vs. AD |
| Accuracy (%) | 72.26 | 72.42 | 90.90 |
| F1-score (%) | 72.15 | 72.37 | 90.88 |
| **Top 8 Features** | | | |
| | CU vs. LMCI | LMCI vs. AD | CU vs. AD |
| Accuracy (%) | 72.34 | 71.60 | 91.40 |
| F1-score (%) | 72.21 | 71.59 | 91.38 |

B) K-fold=10

| All Features | | | |
|---|---|---|---|
| All Features | CU vs. LMCI | LMCI vs. AD | CU vs. AD |
| Acc. | 72.15 | 71.45 | 90.62 |
| F1-score | 71.92 | 71.03 | 90.60 |
| **Top 8 Features** | | | |
| Top 8 Features | CU vs. LMCI | LMCI vs. AD | CU vs. AD |
| Acc. | 71.19 | 70.49 | 91.86 |
| F1-score | 70.98 | 70.15 | 91.81 |

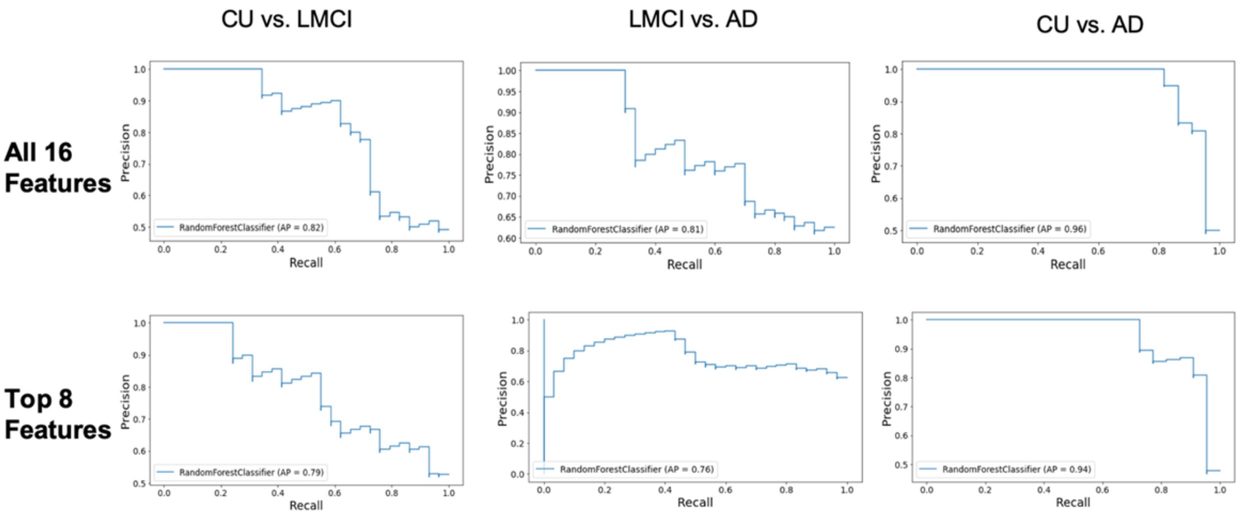


**Supplementary Figure 3.** Comparison of precision recall (PR) curves between all 16 biomarker features (top) and the tope 8 biomarker features (bottom) from the three diagnosis participant group comparisons: cognitively unimpaired (CU) vs late mild cognitive impairment (LMCI), LCMI vs Alzheimer's disease (AD), and CU vs AD. The Average of Precision (AP) is calculated to see the average precision of each model under different possible thresholds.

**Supplementary Table 2. Accuracy results of all features in Gradient Tree Boosting model from A) three-, B) five-, and C) ten-fold cross validation.**

A) K-fold =3

| All Features | | | |
|---|---|---|---|
| | CU vs. LMCI | LMCI vs. AD | CU vs. AD |
| Accuracy (%) | 70.16 | 70.41 | 91.47 |
| F1-score (%) | 69.97 | 70.34 | 91.44 |

B) K-fold=5

| All Features | | | |
|---|---|---|---|
| | CU vs. LMCI | LMCI vs. AD | CU vs. AD |
| Accuracy (%) | 72.30 | 71.26 | 91.87 |
| F1-score (%) | 72.24 | 71.01 | 91.80 |

C) K-fold=10

| All Features | | | |
|---|---|---|---|
| | CU vs. LMCI | LMCI vs. AD | CU vs. AD |
| Accuracy (%) | 70.51 | 70.73 | 92.63 |
| F1-score (%) | 70.29 | 70.36 | 92.61 |

## ACKNOWLEDGEMENT LIST FOR ADNI PUBLICATIONS

The Data and Publications Committee, in keeping with the publication policies adopted by the ADNI Steering Committee, here provide lists for standardized acknowledgement. The list consists of three parts: I. ADNI Infrastructure Investigators and Site Investigators, II. DOD ADNI Infrastructure Investigators and Site Investigators and III. ADNI Depression Infrastructure Investigators and Site Investigators. Infrastructure Investigators represent the names responsible for leadership and infrastructure. Site Investigators represent the names of individuals at each recruiting site. All papers, including methodological papers, should have an acknowledgement list that consists of Infrastructure Investigators plus the FULL list.

### I. ADNI I, GO, II and III

**Part A: Leadership and Infrastructure**

**Principal Investigator**

| | |
|---|---|
| Michael W. Weiner, MD | UC San Francisco |

**ATRI PI and Director of Coordinating Center Clinical Core**

| | |
|---|---|
| Paul Aisen, MD | University of Southern California |

**Executive Committee**

| | |
|---|---|
| Michael Weiner, MD | UC San Francisco |
| Paul Aisen, MD | University of Southern California |
| Ronald Petersen, MD, PhD | Mayo Clinic, Rochester |
| Clifford R. Jack, Jr., MD | Mayo Clinic, Rochester |
| William Jagust, MD | UC Berkeley |
| John Q. Trojanowki, MD, PhD | U Pennsylvania |
| Arthur W. Toga, PhD | USC |
| Laurel Beckett, PhD | UC Davis |
| Robert C. Green, MD, MPH | Brigham and Women’s Hospital/Harvard Medical School |
| Andrew J. Saykin, PsyD | Indiana University |
| John Morris, MD | Washington University St. Louis |
| Leslie M. Shaw | University of Pennsylvania |

**ADNI External Advisory Board (ESAB)**

| | |
|---|---|
| Zaven Khachaturian, PhD | Prevent Alzheimer’s Disease 2020 (Chair) |
| Greg Sorensen, MD | Siemens |
| Maria Carrillo, PhD | Alzheimer’s Association |
| Lew Kuller, MD | University of Pittsburgh |
| Marc Raichle, MD | Washington University St. Louis |
| Steven Paul, MD | Cornell University |
| Peter Davies, MD | Albert Einstein College of Medicine of Yeshiva University |
| Howard Fillit, MD | AD Drug Discovery Foundation |
| Franz Hefti, PhD | Acumen Pharmaceuticals |
| David Holtzman, MD | Washington University St. Louis |
| M. Marcel Mesulam, MD | Northwestern University |
| William Potter, MD | National Institute of Mental Health |
| Peter Snyder, PhD | Brown University |

**ADNI 3 Private Partner Scientific Board (PPSB)**

| | |
|---|---|
| Veronika Logovinsky, MD, PhD | Eli Lilly (Chair) |

**Data and Publications Committee**

| | |
|---|---|
| Robert C. Green, MD, MPH | BWH/HMS (Chair) |

**Resource Allocation Review Committee**

| | |
|---|---|
| Tom Montine, MD, PhD | University of Washington (Chair) |

**Clinical Core Leaders**

| | |
|---|---|
| Ronald Petersen, MD, PhD | Mayo Clinic, Rochester (Core PI) |
| Paul Aisen, MD | University of Southern California |

**Clinical Informatics and Operations**

| | |
|---|---|
| Gustavo Jimenez, MBS | USC |
| Michael Donohue, PhD | USC |
| Devon Gessert, BS | USC |
| Kelly Harless, BA | USC |
| Jennifer Salazar, MBS | USC |
| Yuliana Cabrera, BS | USC |
| Sarah Walter, MSc | USC |
| Lindsey Hergesheimer, BS | USC |

**Biostatistics Core Leaders and Key Personnel**

| | |
|---|---|
| Laurel Beckett, PhD | UC Davis (Core PI) |
| Danielle Harvey, PhD | UC Davis |
| Michael Donohue, PhD | UC San Diego |

**MRI Core Leaders and Key Personnel**

| | |
|---|---|
| Clifford R. Jack, Jr., MD | Mayo Clinic, Rochester (Core PI) |
| Matthew Bernstein, PhD | Mayo Clinic, Rochester |
| Nick Fox, MD | University of London |
| Paul Thompson, PhD | UCLA School of Medicine |
| Norbert Schuff, PhD | UCSF MRI |
| Charles DeCArli, MD | UC Davis |
| Bret Borowski, RT | Mayo Clinic |
| Jeff Gunter, PhD | Mayo Clinic |
| Matt Senjem, MS | Mayo Clinic |
| Prashanthi Vemuri, PhD | Mayo Clinic |
| David Jones, MD | Mayo Clinic |
| Kejal Kantarci | Mayo Clinic |
| Chad Ward | Mayo Clinic |

**PET Core Leaders and Key Personnel**

| | |
|---|---|
| William Jagust, MD | UC Berkeley (Core PI) |
| Robert A. Koeppe, PhD | University of Michigan |
| Norm Foster, MD | University of Utah |
| Eric M. Reiman, MD | Banner Alzheimer's Institute |
| Kewei Chen, PhD | Banner Alzheimer's Institute |
| Chet Mathis, MD | University of Pittsburgh |

| | |
|---|---|
| Susan Landau, PhD | UC Berkeley |

**Neuropathology Core Leaders**

| | |
|---|---|
| John C. Morris, MD | Washington University St. Louis |
| Nigel J. Cairns, PhD, FRCPath | Washington University St. Louis |
| Erin Franklin, MS, CCRP | Washington University St. Louis |
| Lisa Taylor-Reinwald, BA, HTL (ASCP) – Past Investigator | Washington University St. Louis |

**Biomarkers Core Leaders and Key Personnel**

| | |
|---|---|
| Leslie M. Shaw, PhD | UPenn School of Medicine |
| John Q. Trojanowki, MD, PhD | UPenn School of Medicine |
| Virginia Lee, PhD, MBA | UPenn School of Medicine |
| Magdalena Korecka, PhD | UPenn School of Medicine |
| Michal Figurski, PhD | UPenn School of Medicine |

**Informatics Core Leaders and Key Personnel**

| | |
|---|---|
| Arthur W. Toga, PhD | USC (Core PI) |
| Karen Crawford | USC |
| Scott Neu, PhD | USC |

**Genetics Core Leaders and Key Personnel**

| | |
|---|---|
| Andrew J. Saykin, PsyD | Indiana University |
| Tatiana M. Foroud, PhD | Indiana University |
| Steven Potkin, MD UC | UC Irvine |
| Li Shen, PhD | Indiana University |
| Kelley Faber, MS, CCRC | Indiana University |
| Sungeun Kim, PhD | Indiana University |
| Kwangsik Nho, PhD | Indiana University |

**Initial Concept Planning & Development**

| | |
|---|---|
| Michael W. Weiner, MD | UC San Francisco |
| Lean Thal, MD | UC San Diego |
| Zaven Khachaturian, PhD | Prevent Alzheimer's Disease 2020 |

**Early Project Proposal Development**

| | |
|---|---|
| Leon Thal, MD | UC San Diego |
| Neil Buckholtz | National Institute on Aging |
| Michael W. Weiner, MD | UC San Francisco |
| Peter J. Snyder, PhD | Brown University |
| William Potter, MD | National Institute of Mental Health |
| Steven Paul, MD | Cornell University |
| Marilyn Albert, PhD | Johns Hopkins University |
| Richard Frank, MD, PhD | Richard Frank Consulting |
| Zaven Khachaturian, PhD | Prevent Alzheimer's Disease 2020 |

**NIA**

| | |
|---|---|
| John Hsiao, MD | National Institute on Aging |

**Part B: Investigators By Site**

**Oregon Health & Science University:**
Joseph Quinn, MD
Lisa C. Silbert, MD
Betty Lind, BS
Jeffrey A. Kaye, MD, A. – Past Investigator
Raina Carter, BA – Past Investigator
Sara Dolen, BS – Past Investigator

**University of Southern California:**
Lon S. Schneider, MD
Sonia Pawluczyk, MD
Mauricio Becerra, BS
Liberty Teodoro, RN
Bryan M. Spann, DO, PhD – Past Investigator

**University of California – San Diego:**
James Brewer, MD, PhD
Helen Vanderswag, RN
Adam Fleisher, MD – Past Investigator

**University of Michigan:**
Jaimie Ziolkowski, MA, BS, TLLP
Judith L. Heidebrink, MD, MS
Joanne L. Lord, LPN, BA, CCRC – Past Investigator

**Mayo Clinic, Rochester:**
Ronald Petersen, MD, PhD
Sara S. Mason, RN
Colleen S. Albers, RN
David Knopman, MD
Kris Johnson, RN – Past Investigator

**Baylor College of Medicine:**
Javier Villanueva-Meyer, MD
Valory Pavlik, PhD
Nathaniel Pacini, MA
Ashley Lamb, MA
Joseph S. Kass, MD, LD, FAAN
Rachelle S. Doody, MD, PhD – Past Investigator
Victoria Shibley, MS – Past Investigator
Munir Chowdhury, MBBS, MS – Past Investigator
Susan Rountree, MD – Past Investigator
Mimi Dang, MD – Past Investigator

**Columbia University Medical Center:**
Yaakov Stern, PhD
Lawrence S. Honig, MD, PhD
Karen L. Bell, MD
Randy Yeh, MD

**Washington University, St. Louis:**
Beau Ances, MD, PhD, MSc
John C. Morris, MD
David Winkfield, BS
Maria Carroll, RN, MSN, GCNS-BC
Angela Oliver, RN, BSN, MSG
Mary L. Creech, RN, MSW – Past Investigator
Mark A. Mintun, MD – Past Investigator
Stacy Schneider, APRN, BC, GNP – Past Investigator

**University of Alabama - Birmingham:**
Daniel Marson, JD, PhD
David Geldmacher, MD
Marissa Natelson Love, MD
Randall Griffith, PhD, ABPP – Past Investigator
David Clark, MD – Past Investigator
John Brockington, MD – Past Investigator

**Mount Sinai School of Medicine:**
Hillel Grossman, MD
Effie Mitsis, PhD – Past Investigator

**Rush University Medical Center:**
Raj C. Shah, MD
Melissa Lamar, PhD
Patricia Samuels

**Wien Center:**
Ranjan Duara, MD
Maria T. Greig-Custo, MD
Rosemarie Rodriguez, PhD

**Johns Hopkins University:**
Marilyn Albert, PhD
Chiadi Onyike, MD
Daniel D'Agostino II, BS
Stephanie Kielb, BS – Past Investigator

**New York University:**
Martin Sadowski, MD, PhD
Mohammed O. Sheikh, MD
Jamika Singleton-Garvin, CCRP
Anaztasia Ulysse
Mrunalini Gaikwad

**Duke University Medical Center:**
P. Murali Doraiswamy, MBBS, FRCP
Jeffrey R. Petrella, MD
Olga James, MD
Salvador Borges-Neto, MD
Terence Z. Wong, MD – Past Investigator
Edward Coleman – Past Investigator

**University of Pennsylvania:**
Jason H. Karlawish, MD
David A. Wolk, MD
Sanjeev Vaishnavi, MD
Christopher M. Clark, MD – Past Investigator
Steven E. Arnold, MD – Past Investigator

**University of Kentucky:**
Charles D. Smith, MD
Greg Jicha, MD
Peter Hardy, PhD
Riham El Khouli, MD
Elizabeth Oates, MD
Gary Conrad, MD

**University of Pittsburgh:**
Oscar L. Lopez, MD
MaryAnn Oakley, MA
Donna M. Simpson, CRNP, MPH

**University of Rochester Medical Center:**
Anton P. Porsteinsson, MD
Kim Martin, RN
Nancy Kowalksi, MS, RNC
Melanie Keltz, RN
Bonnie S. Goldstein, MS, NP – Past Investigator
Kelly M. Makino, BS – Past Investigator
M. Saleem Ismail, MD – Past Investigator
Connie Brand, RN – Past Investigator

**University of California Irvine IMIND:**
Gaby Thai, MD
Aimee Pierce, MD
Beatriz Yanez, RN
Elizabeth Sosa, PhD
Megan Witbracht, PhD

**University of Texas Southwestern Medical School:**
Kyle Womack, MD
Dana Mathews, MD, PhD
Mary Quiceno, MD

**Emory University:**
Allan I. Levey, MD, PhD
James J. Lah, MD, PhD
Janet S. Cellar, DNP, PMHCNS-BC

**University of Kansas, Medical Center:**
Jeffrey M. Burns, MD
Russell H. Swerdlow, MD
William M. Brooks, PhD

**University of California, Los Angeles:**
Ellen Woo, PhD
Daniel H.S. Silverman, MD, PhD
Edmond Teng, MD, PhD
Sarah Kremen, MD
Liana Apostolova, MD – Past Investigator
Kathleen Tingus, PhD – Past Investigator
Po H. Lu, PsyD – Past Investigator
George Bartzokis, MD – Past Investigator

**Mayo Clinic, Jacksonville:**
Neill R Graff-Radford, MBBCH, FRCP (London)
Francine Parfitt, MSH, CCRC
Kim Poki-Walker, BA

**Indiana University:**
Martin R. Farlow, MD
Ann Marie Hake, MD
Brandy R. Matthews, MD – Past Investigator
Jared R. Brosch, MD
Scott Herring, RN, CCRC

**Yale University School of Medicine:**
Christopher H. van Dyck, MD
Richard E. Carson, PhD
Pradeep Varma, MD

**McGill Univ., Montreal-Jewish General Hospital:**
Howard Chertkow, MD
Howard Bergman, MD
Chris Hosein, MEd

**Sunnybrook Health Sciences, Ontario:**
Sandra Black, MD, FRCPC
Bojana Stefanovic, PhD
Chris (Chinthaka) Heyn, BSC, PhD, MD, FRCPC

**U.B.C. Clinic for AD & Related Disorders:**
Ging-Yuek Robin Hsiung, MD, MHSc, FRCPC
Benita Mudge, BS
Vesna Sossi, PhD
Howard Feldman, MD, FRCPC – Past Investigator
Michele Assaly, MA – Past Investigator

**Cognitive Neurology - St. Joseph's, Ontario:**
Elizabeth Finger, MD
Stephen Pasternack, MD, PhD
William Pavlosky, MD
Irina Rachinsky, MD – Past Investigator
Dick Drost, PhD – Past Investigator
Andrew Kertesz, MD – Past Investigator

**Cleveland Clinic Lou Ruvo Center for Brain Health:**
Charles Bernick, MD, MPH
Donna Munic, PhD

**Northwestern University:**
Marek-Marsel Mesulam, MD
Emily Rogalski, PhD
Kristine Lipowski, MA
Sandra Weintraub, PhD
Borna Bonakdarpour, MD
Diana Kerwin, MD – Past Investigator
Chuang-Kuo Wu, MD, PhD – Past Investigator
Nancy Johnson, PhD – Past Investigator

**Premiere Research Inst (Palm Beach Neurology):**
Carl Sadowsky, MD
Teresa Villena, MD

**Georgetown University Medical Center:**
Raymond Scott Turner, MD, PhD
Kathleen Johnson, NP
Brigid Reynolds, NP

**Brigham and Women's Hospital:**
Reisa A. Sperling, MD
Keith A. Johnson, MD
Gad A. Marshall, MD

**Stanford University:**
Jerome Yesavage, MD
Joy L. Taylor, PhD
Steven Chao, MD, PhD
Barton Lane, MD – Past Investigator
Allyson Rosen, PhD – Past Investigator
Jared Tinklenberg, MD – Past Investigator

**Banner Sun Health Research Institute:**
Edward Zamrini, MD
Christine M. Belden, PsyD
Sherye A. Sirrel, CCRC

**Boston University:**
Neil Kowall, MD
Ronald Killiany, PhD
Andrew E. Budson, MD
Alexander Norbash, MD – Past Investigator
Patricia Lynn Johnson, BA – Past Investigator

**Howard University:**
Thomas O. Obisesan, MD, MPH
Ntekim E. Oyonumo, MD, PhD
Joanne Allard, PhD
Olu Ogunlana, BPharm

**Case Western Reserve University:**
Alan Lerner, MD
Paula Ogrocki, PhD
Curtis Tatsuoka, PhD
Parianne Fatica, BA, CCRC

**University of California, Davis – Sacramento:**
Evan Fletcher, PhD
Pauline Maillard, PhD
John Olichney, MD
Charles DeCarli, MD
Owen Carmichael, PhD – Past Investigator

**Neurological Care of CNY:**
Smita Kittur, MD – Past Investigator

**Parkwood Institute:**
Michael Borrie, MB ChB
T-Y Lee, PhD
Dr Rob Bartha, PhD

**University of Wisconsin:**
Sterling Johnson, PhD
Sanjay Asthana, MD

Cynthia M. Carlsson, MD, MS

**Banner Alzheimer's Institute:**
Pierre Tariot, MD
Anna Burke, MD
Joel Hetelle, BS
Kathryn DeMarco, BS
Nadira Trncic, MD, PhD, CCRC – Past Investigator
Adam Fleisher, MD – Past Investigator
Stephanie Reeder, BA – Past Investigator

**Dent Neurologic Institute:**
Vernice Bates, MD
Horacio Capote, MD
Michelle Rainka, PharmD, CCRP

**Ohio State University:**
Douglas W. Scharre, MD
Maria Kataki, MD, PhD
Rawan Tarawneh, MD

**Albany Medical College:**
Earl A. Zimmerman, MD
Dzintra Celmins, MD
David Hart, MD

**Hartford Hospital, Olin Neuropsychiatry Research Center:**
Godfrey D. Pearlson, MD
Karen Blank, MD
Karen Anderson, RN

**Dartmouth-Hitchcock Medical Center:**
Laura A. Flashman, PhD
Marc Seltzer, MD
Mary L. Hynes, RN, MPH
Robert B. Santulli, MD – Past Investigator

**Wake Forest University Health Sciences:**
Kaycee M. Sink, MD, MAS
Mia Yang, MD
Akiva Mintz, MD, PhD

**Rhode Island Hospital:**
Brian R. Ott, MD
Geoffrey Tremont, PhD
Lori A. Daiello, Pharm.D, ScM

**Butler Hospital:**
Courtney Bodge, PhD
Stephen Salloway, MD, MS
Paul Malloy, PhD
Stephen Correia, PhD
Athena Lee, PhD

**UC San Francisco:**
Howard J. Rosen, MD
Bruce L. Miller, MD
David Perry, MD

**Medical University South Carolina:**
Jacobo Mintzer, MD, MBA
Kenneth Spicer, MD, PhD
David Bachman, MD

**St. Joseph's Health Care:**
Elizabeth Finger, MD
Stephen Pasternak, MD
Irina Rachinsky, MD
John Rogers, MD
Andrew Kertesz, MD – Past Investigator
Dick Drost, MD – Past Investigator

**Nathan Kline Institute**
Nunzio Pomara, MD
Raymundo Hernando, MD
Antero Sarrael, MD

**University of Iowa College of Medicine**
Delwyn D. Miller, PharmD, MD
Karen Ekstam Smith, RN
Hristina Koleva, MD
Ki Won Nam, MD
Hyungsub Shim, MD
Susan K. Schultz, MD – Past Investigator

**Cornell University**
Norman Relkin, MD, PhD
Gloria Chiang, MD
Michael Lin, MD
Lisa Ravdin, PhD

**University of South Florida: USF Health Byrd Alzheimer's Institute**
Amanda Smith, MD
Christi Leach, MD
Balebail Ashok Raj, MD – Past Investigator
Kristin Fargher, MD – Past Investigator

**DOD ADNI**

**Part A: Leadership and Infrastructure**

**Principal Investigator**

| | |
|---|---|
| Michael W. Weiner, MD | University of California, San Francisco |

**ATRI PI and Director of Coordinating Center Clinical Core**

| | |
|---|---|
| Paul Aisen, MD | University of Southern California |

**Executive Committee**

| | |
|---|---|
| Michael Weiner, MD | UC San Francisco |
| Paul Aisen, MD | University of Southern California |
| Ronald Petersen, MD, PhD | Mayo Clinic, Rochester |
| Robert C. Green, MD, MPH | Brigham and Women's Hospital/ Harvard Medical School |
| Danielle Harvey, PhD | UC Davis |
| Clifford R. Jack, Jr., MD | Mayo Clinic, Rochester |
| William Jagust, MD | UC Berkeley |
| John C. Morris, MD | Washington University St. Louis |
| Andrew J. Saykin, PsyD | Indiana University |
| Leslie M. Shaw, PhD | Perelman School of Medicine, UPenn |
| Arthur W. Toga, PhD | USC |
| John Q. Trojanowki, MD, PhD | Perelman School of Medicine, University of Pennsylvania |

**Psychological Evaluation/PTSD Core**

| | |
|---|---|
| Thomas Neylan, MD | UC San Francisco |

**Traumatic Brain Injury/TBI Core**

| | |
|---|---|
| Jordan Grafman, PhD | Rehabilitation Institute of Chicago, Feinberg School of Medicine, Northwestern University |

**Data and Publication Committee (DPC)**

| | |
|---|---|
| Robert C. Green, MD, MPH | BWH/HMS (Chair) |

**Resource Allocation Review Committee**

| | |
|---|---|
| Tom Montine, MD, PhD | University of Washington (Chair) |

**Clinical Core Leaders**

| | |
|---|---|
| Michael Weiner MD | Core PI |
| Ronald Petersen, MD, PhD | Mayo Clinic, Rochester (Core PI) |
| Paul Aisen, MD | University of Southern California |

**Clinical Informatics and Operations**

| | |
|---|---|
| Gustavo Jimenez, MBS | USC |
| Michael Donohue, PhD | USC |
| Devon Gessert, BS | USC |

| | |
|---|---|
| Kelly Harless, BA | USC |
| Jennifer Salazar, MBS | USC |
| Yuliana Cabrera, BS | USC |
| Sarah Walter, MSc | USC |
| Lindsey Hergesheimen, BS | USC |

**San Francisco Veterans Affairs Medical Center**

| | |
|---|---|
| Thomas Neylan, MD | UC San Francisco |
| Jacqueline Hayes | UC San Francisco |
| Shannon Finley | UC San Francisco |

**Biostatistics Core Leaders and Key Personnel**

| | |
|---|---|
| Danielle Harvey, PhD | UC Davis (Core PI) |
| Michael Donohue, PhD | UC San Diego |

**MRI Core Leaders and Key Personnel**

| | |
|---|---|
| Clifford R. Jack, Jr., MD | Mayo Clinic, Rochester (Core PI) |
| Matthew Bernstein, PhD | Mayo Clinic, Rochester |
| Bret Borowski, RT | Mayo Clinic |
| Jeff Gunter, PhD | Mayo Clinic |
| Matt Senjem, MS | Mayo Clinic |
| Kejal Kantarci | Mayo Clinic |
| Chad Ward | Mayo Clinic |

**PET Core Leaders and Key Personnel**

| | |
|---|---|
| William Jagust, MD | UC Berkeley (Core PI) |
| Robert A. Koeppe, PhD | University of Michigan |
| Norm Foster, MD | University of Utah |
| Eric M. Reiman, MD | Banner Alzheimer's Institute |
| Kewei Chen, PhD | Banner Alzheimer's Institute |
| Susan Landau, PhD | UC Berkeley |

**Neuropathology Core Leaders**

| | |
|---|---|
| John C. Morris, MD | Washington University St. Louis |
| Nigel J. Cairns, PhD, FRCPath | Washington University St. Louis |
| Erin Householder, MS | Washington University St. Louis |

**Biomarkers Core Leaders and Key Personnel**

| | |
|---|---|
| Leslie M. Shaw, PhD | Perelman School of Medicine, UPenn |
| John Q. Trojanowki, MD, PhD | Perelman School of Medicine, UPenn |
| Virginia Lee, PhD, MBA | Perelman School of Medicine, UPenn |
| Magdalena Korecka, PhD | Perelman School of Medicine, UPenn |
| Michal Figurski, PhD | Perelman School of Medicine, UPenn |

**Informatics Core Leaders and Key Personnel**

| | |
|---|---|
| Arthur W. Toga, PhD | USC (Core PI) |
| Karen Crawford | USC |
| Scott Neu, PhD | USC |

**Genetics Core Leaders and Key Personnel**

| | |
|---|---|
| Andrew J. Saykin, PsyD | Indiana University |
| Tatiana M. Foroud, PhD | Indiana University |
| | |
| Steven Potkin, MD UC | UC Irvine |
| Li Shen, PhD | Indiana University |
| Kelley Faber, MS, CCRC | Indiana University |
| Sungeun Kim, PhD | Indiana University |
| Kwangsik Nho, PhD | Indiana University |

**Initial Concept Planning & Development**

| | |
|---|---|
| Michael W. Weiner, MD | UC San Francisco |
| Karl Friedl | Department of Defense (retired) |

**Part B: Investigators By Site**

**University of Southern California:**
Lon S. Schneider, MD, MS
Sonia Pawluczyk, MD
Mauricio Becerra

**University of California, San Diego:**
James Brewer, MD, PhD
Helen Vanderswag, RN

**Columbia University Medical Center:**
Yaakov Stern, PhD
Lawrence S. Honig, MD, PhD
Karen L. Bell, MD

**Rush University Medical Center:**
Debra Fleischman, Ph.D.
Konstantinos Arfanakis, Ph.D.
Raj C. Shah, M.D.

**Wien Center:**
Dr. Ranjan Duara MD PI
Dr. Daniel Varon MD Co-PI
Maria T Greig HP Coordinator

**Duke University Medical Center:**
P. Murali Doraiswamy, MBBS
Jeffrey R. Petrella, MD
Olga James, MD

**University of Rochester Medical Center:**
Anton P. Porsteinsson, MD (director)
Bonnie Goldstein, MS, NP (coordinator)
Kimberly S. Martin, RN

**University of California, Irvine:**
Steven G. Potkin, MD
Adrian Preda, MD
Dana Nguyen, PhD

**Medical University South Carolina:**
Jacobo Mintzer, MD, MBA
Dino Massoglia, MD, PhD
Olga Brawman-Mintzer, MD

**Premiere Research Inst (Palm Beach Neurology):**
Carl Sadowsky, MD
Walter Martinez, MD
Teresa Villena, MD

**University of California, San Francisco:**
William Jagust MD
Susan Landau PhD
Howard Rosen, MD
David Perry

**Georgetown University Medical Center:**
Raymond Scott Turner, MD, PhD
Kelly Behan
Brigid Reynolds, NP

**Brigham and Women's Hospital:**
Reisa A. Sperling, MD
Keith A. Johnson, MD
Gad Marshall, MD

**Banner Sun Health Research Institute:**
Marwan N. Sabbagh, MD
Sandra A. Jacobson, MD

Sherye A. Sirrel, MS, CCRC

**Howard University:**
Thomas O. Obisesan, MD, MPH
Saba Wolday, MSc
Joanne Allard, PhD

**University of Wisconsin:**
Sterling C. Johnson, Ph.D.
J. Jay Fruehling, M.A.
Sandra Harding, M.S.

**University of Washington:**
Elaine R. Peskind, MD
Eric C. Petrie, MD, MS

Gail Li, MD, PhD

**Stanford University:**
Jerome A. Yesavage, MD
Joy L. Taylor, PhD
Ansgar J. Furst, PhD
Steven Chao, M.D.

**Cornell University:**
Norman Relkin, MD, PhD
Gloria Chiang, MD
Lisa Ravdin, PhD

**ADNI Depression**

**Part A: Leadership and Infrastructure**

**Principal Investigator**

| | |
|---|---|
| Scott Mackin, PhD | University of California, San Francisco |

**ATRI PI and Director of Coordinating Center Clinical Core**

| | |
|---|---|
| Paul Aisen, MD | University of Southern California |
| Rema Raman, PhD | University of Southern California |

**Executive Committee**

| | |
|---|---|
| Scott Mackin, PhD | UC San Francisco |
| Michael Weiner, MD | UC San Francisco |
| Paul Aisen, MD | University of Southern California |
| Rema Raman, PhD | University of Southern California |
| Clifford R. Jack, Jr., MD | Mayo Clinic, Rochester |
| Susan Landau, PhD | UC Berkeley |
| Andrew J. Saykin, PsyD | Indiana University |
| Arthur W. Toga, PhD | University of Southern California |
| Charles DeCarli, MD | UC Davis |
| Robert A. Koeppe, PhD | University of Michigan |

**Data and Publication Committee (DPC)**

| | |
|---|---|
| Robert C. Green, MD, MPH | BWH/HMS (Chair) |
| Erin Drake, MA | BWM/HMS (Director) |

**Clinical Core Leaders**

| | |
|---|---|
| Michael Weiner MD | Core PI |
| Paul Aisen, MD | University of Southern California |
| Rema Raman, PhD | University of Southern California |
| Mike Donohue, PhD | University of Southern California |

**Clinical Informatics, Operations and Regulatory Affairs**

| | |
|---|---|
| Gustavo Jimenez, MBS | USC |
| Devon Gessert, BS | USC |
| Kelly Harless, BA | USC |
| Jennifer Salazar, MBS | USC |
| Yuliana Cabrera, BS | USC |
| Sarah Walter, MSc | USC |
| Lindsey Hergesheimer, BS | USC |
| Elizabeth Shaffer, BS | |

**Psychiatry Site Leaders and Key Personnel**

| | |
|---|---|
| Scott Mackin, PhD | UC San Francisco |
| Craig Nelson, MD | UC San Francisco |
| David Bickford, BA | UC San Francisco |
| Meryl Butters, PhD | University of Pittsburgh |
| Michelle Zmuda, MA | University of Pittsburgh |

**MRI Core Leaders and Key Personnel**

| | |
|---|---|
| Clifford R. Jack, Jr., MD | Mayo Clinic, Rochester (Core PI) |
| Matthew Bernstein, PhD | Mayo Clinic, Rochester |
| Bret Borowski, RT | Mayo Clinic, Rochester |
| Jeff Gunter, PhD | Mayo Clinic, Rochester |
| Matt Senjem, MS | Mayo Clinic, Rochester |
| Kejal Kantarci, MD | Mayo Clinic, Rochester |
| Chad Ward, BA | Mayo Clinic, Rochester |
| Denise Reyes, BS | Mayo Clinic, Rochester |

**PET Core Leaders and Key Personnel**

| | |
|---|---|
| Robert A. Koeppe, PhD | University of Michigan |
| Susan Landau, PhD | UC Berkeley |

**Informatics Core Leaders and Key Personnel**

| | |
|---|---|
| Arthur W. Toga, PhD | USC (Core PI) |
| Karen Crawford | USC |
| Scott Neu, PhD | USC |

**Genetics Core Leaders and Key Personnel**

| | |
|---|---|
| Andrew J. Saykin, PsyD | Indiana University |
| Tatiana M. Foroud, PhD | Indiana University |
| Kelley M. Faber, MS, CCRC | Indiana University |
| Kwangsik Nho, PhD | Indiana University |
| Kelly N. Nudelman | Indiana University |

**Part B: Investigators By Site**

**University of California, San Francisco:**
Scott Mackin, PhD
Howard Rosen, MD
Craig Nelson, MD
David Bickford, BA
Yiu Ho Au, BA
Kelly Scherer, BS
Daniel Catalinotto, BA
Samuel Stark, BA
Elise Ong, BA
Dariella Fernandez, BA

**University of Pittsburgh:**
Meryl Butters, PhD
Michelle Zmuda, MA
Oscar L. Lopez, MD
MaryAnn Oakley, MA
Donna M. Simpson, CRNP, MPH
