# Supplementary material for: β-amyloid and tau drive early Alzheimer’s disease decline while glucose hypometabolism drives late decline
Source: Commun Biol. 2020 Jul 6;3:352. doi: 10.1038/s42003-020-1079-x (PMC7338410; doi:10.1038/s42003-020-1079-x)
Supplement: Supplementary file 2 — Reporting Summary [file 42003_2020_1079_MOESM2_ESM.pdf]

nature research

Corresponding author(s): Ai-Ling Lin, PhD

Last updated by author(s): Apr 6, 2020

Reporting Summary

Nature Research wishes to improve the reproducibility of the work that we publish. This form provides structure for consistency and transparency in reporting. For further information on Nature Research policies, see Authors & Referees and the Editorial Policy Checklist.

Statistics

For all statistical analyses, confirm that the following items are present in the figure legend, table legend, main text, or Methods section.

n/a	Confirmed	
☐	☒	The exact sample size (*n*) for each experimental group/condition, given as a discrete number and unit of measurement
☐	☒	A statement on whether measurements were taken from distinct samples or whether the same sample was measured repeatedly
☐	☒	The statistical test(s) used AND whether they are one- or two-sided<br>*Only common tests should be described solely by name; describe more complex techniques in the Methods section.*
☐	☒	A description of all covariates tested
☐	☒	A description of any assumptions or corrections, such as tests of normality and adjustment for multiple comparisons
☐	☒	A full description of the statistical parameters including central tendency (e.g. means) or other basic estimates (e.g. regression coefficient) AND variation (e.g. standard deviation) or associated estimates of uncertainty (e.g. confidence intervals)
☐	☒	For null hypothesis testing, the test statistic (e.g. *F*, *t*, *r*) with confidence intervals, effect sizes, degrees of freedom and *P* value noted<br>*Give P values as exact values whenever suitable.*
☒	☐	For Bayesian analysis, information on the choice of priors and Markov chain Monte Carlo settings
☐	☒	For hierarchical and complex designs, identification of the appropriate level for tests and full reporting of outcomes
☐	☒	Estimates of effect sizes (e.g. Cohen's *d*, Pearson's *r*), indicating how they were calculated

Our web collection on statistics for biologists contains articles on many of the points above.

Software and code

Policy information about availability of computer code

Data collection	Study data were obtained from the ADNI database, a longitudinal multicenter study designed to develop clinical, imaging, genetic, and biochemical biomarkers for the early detection and tracking of Alzheimer’s disease. The dataset was downloaded from the ADNI2 dataset within the ADNI database since these data contained all the biomarkers of interest for the present study. The data were merged from five subset datasets within the ADNI2 dataset to achieve a final dataset for analysis consisting of demographic information, structural MRI volumes, FDG-PET SUVs, amyloid-PET SUVs, White Matter Hyperintensities, and CSF-ptau measurements. Age, gender, education, APOE4 carrier status, cognitive scores, and diagnosis and the structural MRI variables of ventricle volume, whole brain volume, entorhinal cortex volume, and hippocampal volume were extracted from the ADNIMERGE subset dataset. FDG-Angular, FDG-Temporal, and FDG-CingulumPost were extracted from the UC Berkeley FDG subset dataset. Aβ-Frontal, Aβ-Cingulate, Aβ-Parietal, Aβ-Temporal, Aβ-Precuneus, and Aβ-Hippocampus were extracted from the UC Berkeley AV45 subset dataset. Gray matter volume, White matter volume, and White matter hyperintensity were extracted from the UC Davis White Matter Hyperintensity Volumes subset dataset. pTau concentration was extracted from the UPENN CSF Biomarkers Elecsys subset dataset. Missing values were imputed by selecting the twenty closest patients based on Euclidean distance with non-missing values in the same group and averaging these values. Most of the missing values appeared in the structural MRI data. Data imputation was performed on patients who had less than three missing values. Patients with three or more missing values were deleted to avoid bias caused by excessive imputation.
Data analysis	To implement the RF model, the scikit-learn library64, a popular Python package for machine learning, was used for all machine learning analyses. The version of scikit-learn is v0.21.3, and the Python environment is Python 3.7. The RF model can be easily accessed by calling sklearn.ensemble.RandomForestClassifier of scikit-learn package.

For manuscripts utilizing custom algorithms or software that are central to the research but not yet described in published literature, software must be made available to editors/reviewers. We strongly encourage code deposition in a community repository (e.g. GitHub). See the Nature Research guidelines for submitting code & software for further information.

Data

Policy information about availability of data

All manuscripts must include a data availability statement. This statement should provide the following information, where applicable:

- Accession codes, unique identifiers, or web links for publicly available datasets
- A list of figures that have associated raw data
- A description of any restrictions on data availability

The datasets analyzed during the current study are available in the Alzheimer's Disease Neuroimaging Initiative (ADNI) respository, http://adni.loni.usc.edu/

Field-specific reporting

Please select the one below that is the best fit for your research. If you are not sure, read the appropriate sections before making your selection.

☒ Life sciences ☐ Behavioural & social sciences ☐ Ecological, evolutionary & environmental sciences

For a reference copy of the document with all sections, see nature.com/documents/nr-reporting-summary-flat.pdf

Life sciences study design

All studies must disclose on these points even when the disclosure is negative.

Sample size	Participant data was extracted from the ADNI database for inclusion in the analysis. Participants were required to have baseline Aβ imaging biomarkers (from 18Florbetapir PET), glucose uptake imaging biomarkers (from 18FDG PET), brain volume imaging biomarkers (from T1-weighted structural MRI), and cognitive testing to be included in the analysis. As tau imaging was not available for most participants in the ADNI database, we used a phosphorylated tau biomarker (pTau) from cerebrospinal fluid (CSF) as a measure of tau levels. These criteria yielded a final sample of 405 participants clinically diagnosed as being either cognitively unimpaired (CU; n = 148) or with late mild cognitive impairment (LMCI; n = 147) or Alzheimer's disease (AD; n = 110)
Data exclusions	Participants with three or more missing values were excluded from the analysis.
Replication	To verify the five-fold validation, we compared the results of accuracy and F1 score from those using three- and ten-fold cross validations.<br><br>To verify the accuracy using ROC, we also performed precision recall (PR) curves calculation.<br><br>We also used the SHapley Additive exPlanations (SHAP) technique to implement an additional feature ranking analysis. In our experiments, we applied the SHAP on Random Forest Regressor, because SHAP has limited function on the Random Forest classifier. Using the regression model as a reference for feature ranking analysis, the SHAP method showed similar feature importance ranking as RF.<br><br>Gradient tree boosting (GTB), another classification method from the scikit-learn package, was used as a comparison for the RF classification method (Supplementary Table 2). The same tree estimators from the RF method were used for GTB with all other default function parameters. The accuracies for the GTB method were similar to the RF method. The accuracy of the GTB classifiers were 72.30%, 71.26%, and 91.87% respectively for CU vs LMCI, LMCI vs AD, and CU vs AD clinical diagnosis. The model was also trained with 3- and 10-fold cross validation for comparison. There were minor difference in the feature rankings estimated using the GTB model as compared to the RF model but the same general patterns hold true: Aβ and pTau are important contributors to the prediction of early AD decline, but neurodegeneration, especially glucose hypometabolism, is a more important predictor of later AD decline.
Randomization	Randomization was not possible. This is a prospective observational cohort study.
Blinding	Blinding was not relevant since participants were not allocated to groups.

Reporting for specific materials, systems and methods

We require information from authors about some types of materials, experimental systems and methods used in many studies. Here, indicate whether each material, system or method listed is relevant to your study. If you are not sure if a list item applies to your research, read the appropriate section before selecting a response.

Materials & experimental systems

n/a	Involved in the study	
☒	☐	Antibodies
☒	☐	Eukaryotic cell lines
☒	☐	Palaeontology
☒	☐	Animals and other organisms
☒	☐	Human research participants
☒	☐	Clinical data

Methods

n/a	Involved in the study	
☒	☐	ChIP-seq
☒	☐	Flow cytometry
☒	☐	MRI-based neuroimaging
